# Ciguatoxins Evoke Potent CGRP Release by Activation of Voltage-Gated Sodium Channel Subtypes Na_V_1.9, Na_V_1.7 and Na_V_1.1

**DOI:** 10.3390/md15090269

**Published:** 2017-08-30

**Authors:** Filip Touska, Simon Sattler, Philipp Malsch, Richard J. Lewis, Peter W. Reeh, Katharina Zimmermann

**Affiliations:** 1 Department of Anesthesiology, Friedrich-Alexander-University Erlangen-Nuremberg, University Hospital Erlangen, Krankenhausstraße 12, 91054 Erlangen, Germany; touska@biomed.cas.cz; 2 Department of Cellular Neurophysiology, Institute of Physiology of the Czech Academy of Sciences, Videnska 1083, 142 20 Prague 4, Czech Republic; 3 Department of Physiology and Pathophysiology, Friedrich-Alexander-University Erlangen-Nuremberg, Universitätsstraße 17, 91054 Erlangen, Germany; simon.sattler@outlook.com (S.S.); philippmalsch@googlemail.com (P.M.); peter.reeh@fau.de (P.W.R.); 4 Institute for Molecular Biosciences, IMB Centre for Pain Research, University of Queensland, 4072 St Lucia, Australia; r.lewis@uq.edu.au

**Keywords:** voltage-gated calcium channels, calcitonin-gene related peptide, tetrodotoxin, TTX, P-CTX-1, TRPA1, TRPC5, TRPM8, ciguatera, neuropathic pain, neurogenic inflammation

## Abstract

Ciguatoxins (CTXs) are marine toxins that cause ciguatera fish poisoning, a debilitating disease dominated by sensory and neurological disturbances that include cold allodynia and various painful symptoms as well as long-lasting pruritus. Although CTXs are known as the most potent mammalian sodium channel activator toxins, the etiology of many of its neurosensory symptoms remains unresolved. We recently described that local application of 1 nM Pacific Ciguatoxin-1 (P-CTX-1) into the skin of human subjects induces a long-lasting, painful axon reflex flare and that CTXs are particularly effective in releasing calcitonin-gene related peptide (CGRP) from nerve terminals. In this study, we used mouse and rat skin preparations and enzyme-linked immunosorbent assays (ELISA) to study the molecular mechanism by which P-CTX-1 induces CGRP release. We show that P-CTX-1 induces CGRP release more effectively in mouse as compared to rat skin, exhibiting EC_50_ concentrations in the low nanomolar range. P-CTX-1-induced CGRP release from skin is dependent on extracellular calcium and sodium, but independent from the activation of various thermosensory transient receptor potential (TRP) ion channels. In contrast, lidocaine and tetrodotoxin (TTX) reduce CGRP release by 53–75%, with the remaining fraction involving L-type and T-type voltage-gated calcium channels (VGCC). Using transgenic mice, we revealed that the TTX-resistant voltage-gated sodium channel (VGSC) Na_V_1.9, but not Na_V_1.8 or Na_V_1.7 alone and the combined activation of the TTX-sensitive VGSC subtypes Na_V_1.7 and Na_V_1.1 carry the largest part of the P-CTX-1-caused CGRP release of 42% and 34%, respectively. Given the contribution of CGRP to nociceptive and itch sensing pathways, our findings contribute to a better understanding of sensory symptoms of acute and chronic ciguatera that may help in the identification of potential therapeutics.

## 1. Introduction

Ciguatera is a neurological disease that is caused by the consumption of toxic tropical and subtropical fish that have bioaccumulated effective levels of ciguatoxins (CTXs). CTXs are cyclic polyether molecules derived from gambiertoxins and originate from certain strains of the dinoflagellate *Gambierdiscus toxicus*. The precursors are oxidized to CTXs in the fish liver by cytochrome enzymes to become the most potent mammalian voltage-gated sodium channel (VGSC) toxins known. The Pacific variants of CTX (e.g., Pacific Ciguatoxin-1, P-CTX-1) are part of three variants and the most abundant and potent with an LD_50_ in mice of 0.25 µg/kg i.p. [1]. Ciguatera is known for causing a myriad of painful and sensory symptoms, including distressing, often persistent sensory disturbances such as perioral and distal paresthesias, dysesthesias, pruritus, headache, asthenia, myalgia, arthralgia and tooth pain [2]. Of these neurological disturbances, temperature dysesthesia, or cold allodynia, a pathophysiological condition where innocuous cold is perceived as painful burning of the skin, is considered pathognomonic and occurs in up to 95% of ciguatera sufferers.

The neurological symptoms are the effect of variable activation of both tetrodotoxin-sensitive (TTXs) and resistant (TTXr) VGSCs depending on the tissue expression of the particular subtypes and the contingent depolarizing effect on other voltage-gated ion channels [3,4,5]. For example, we found that cold allodynia results from the particular activation of a specific subpopulation of sensory neurons that expresses the TTXr VGSC Na_V_1.8, the Transient Receptor Potential Ankyrin 1 (TRPA1) cation channel and the calcitonin gene-related peptide (CGRP) [6]. Our study demonstrated that the activation of VGSCs by P-CTX-1 was sufficient to trigger calcium influx through TRPA1 and to sensitize the sensory neurons to cold, an effect that was absent in sensory neurons derived from TRPA1-deficient mice. Furthermore, we found P-CTX-1 to belong to the most potent compounds to induce a long-lasting axon reflex flare in human skin at a concentration as low as 1 nM. The flare was accompanied by a slowly fading burning pain and followed by transient cold allodynia located in the area of the intracutaneous injection and it occurred most likely due to the potent capacity of P-CTX-1 to induce release of CGRP [7,8].

CGRP is a marker of peptidergic nociceptive neurons and reflects expression of two variants, CGRPα and CGRPβ, which are encoded by separate genes. CGRP is stored in vesicles and its calcium influx-dependent release mediates neurogenic inflammation, including local vasodilation, or can lead to systemic effects via a specific pathway that was found to involve nitroxyl activation of TRPA1 [9,10]. Recently it was found that CGRPα-expressing sensory pathways are required to sense heat, but also itch, a central and long-lasting symptom of ciguatera, and they suppress cold-responsive spinal neurons [11]. Other publications revealed that during chronic inflammatory diseases such as colitis, arthritis, and in migraine release of neuropeptides such as CGRP, but also Substance P (SP) and neuroimmune interactions may contribute critically to the pathogenesis of the diseases [10,12,13].

Neuroimmune interactions may also be crucial for the development of chronic ciguatera, a long-lasting form of ciguatera, which seems to be sustained by chronic activation of the immune system [14]. In fact, with a half-maximally effective concentration of less than 3 nM, P-CTX-1 appears as the most potent compound to release CGRP from mammalian nerve endings known to date [8,15]. Therefore, a more mechanistic insight into how CTXs cause CGRP release from nerve endings could be beneficial for the understanding of sensory symptoms in acute and chronic ciguatera.

To this end we measured CGRP release from isolated rat and mouse hindpaw skin using the ELISA technique. We used transgenic mice lacking TRPA1, TRPV1, TRPC5 and TRPM8 as well as Na_V_1.8, Na_V_1.9 and Advillin-Cre-based Na_V_1.7-deficient mice. In addition we evaluated the effect of blockers of voltage-gated T- and L-type calcium channels (VGCC) as well as TTX and a highly selective small molecule blocker of Na_V_1.1, ICA-121431 [16].

## 2. Material and Methods

### 2.1. Animals, Preparation of Skin Flaps and Solutions

For the release experiments we used skin flaps of the lower hindpaws of young male Wistar rats (70–80 g; *n* = 39) and male C57BL/6J mice (15–35 g; *n* = 136). Additionally, we used different transgenic mice with the genetic background of C57BL/6J which were matched to their respective littermates or C57BL/6J mice when congenic. C57BL/6J mice were obtained from Charles River (Sulzfeld, Germany) and crossed in house. The mice were held in an open-cage facility with a 12 h light-dark cycle at 24 °C room temperature and 40–60% humidity with food and water provided *ad libitum*. We used TRPV1 −/− *n* = 5 and *n* = 6 C57BL/6J control group [17]; TRPA1 −/− and TRPA1 +/+: *n* = 9 each [18]; TRPM8 −/− and TRPM8 +/+: *n* = 7 each [19]; TRPC5 −/− and TRPC5 +/+: *n* = 7 each [20]; Na_V_1.8 −/− and Na_V_1.8 +/+: *n* = 12 each [21]; Na_V_1.9 −/−: *n* = 17 and Na_V_1.9 +/+: *n* = 12 [22]; Na_V_1.7 −/− (floxed Na_V_1.7—Advillin-Cre-null mutant mice) and C57BL/6J: *n* = 10 each [23]. The animals were genotyped according to previously published procedures, as indicated in the respective publication of each transgenic mouse strain cited in the previous paragraph. The animals were sacrificed by exposure to a rising CO_2_ concentration and the hairy skin of both hindpaws was subcutaneously excised sparing the cutaneous nerve branches. The preparation started at knee level and excluded the toes and it spared larger vessels, and saphenous and peroneal nerve stems. The obtained murine skin flaps had an average weight of 0.10 g while the average weight of rat skin flaps was 0.28 g. They were wrapped around acrylic glass rods with the corium side exposed to the surrounding solution and were fixed with surgical silk threads. The mounted skin flaps were then washed for 30 min in carbogen-gassed synthetic interstitial fluid (SIF, pH 7.4) which consisted of (mM): 107.8 NaCl, 3.5 KCl, 0.69 MgSO_4_, 26.2 NaHCO_3_, 1.67 NaH_2_PO_4_, 1.53 CaCl_2_, 9.64 sodium gluconate, 5.55 glucose and 7.6 sucrose. SIF free of extracellular sodium had no NaCl, NaHCO_3_, NaH_2_PO_4_ and sodium gluconate and contained instead (in mM) 108.8 choline chloride, 1 HEPES and 62.49 sucrose; Ca^2+^-free SIF was prepared without sucrose and CaCl_2_ and contained 10 mM EGTA. SIF with 60 mM KCl had NaCl reduced to 47.8 mM.

### 2.2. Stimulation Procedure and Compounds

After the initial 30 min wash-out in SIF, the skin flaps were placed for 5 min each into pre-warmed glass tubes mounted in a shaking bath at 32 °C. One day prior to the experiments, the tubes were treated with Sigmacote© (Sigma-Aldrich, Taufkirchen, Germany) to prevent adhesion of CGRP and P-CTX-1 to the glass surface. Sigmacote^®^ was refreshed after each 10 experiments. All tubes were filled with 0.7 mL of SIF or stimulation solution containing various concentrations of P-CTX-1 or drugs. Chemical treatment was performed during the second or third incubation step, depending on the experiment. Variations of drug application are described in the text. If not stated differently stimulation compounds were dissolved in standard SIF.

P-CTX-1 (>95% purity) was isolated from moray eel (*Gymnothorax javanicus*) liver as previously described [24], stored as a 1 µM concentrated stock in 50% methanol/50% *aqua destillata* and diluted in SIF in the presence of 0.1–0.3% bovine serum albumin (BSA) to avoid loss to plastic. All other reagents were from Sigma-Aldrich unless otherwise stated. We used the following drugs: tetrodotoxin citrate (purchased from Ascent Scientific, now part of AbcamBiochemicals, Cambridge, UK) and prepared as stock solution at a concentration of 1 mM. TTX was used at a final concentration of 1, 10 or 100 µM. Lidocaine was used at a final concentration of 1 mM and kept at a stock solution of 100 mM. Mibefradil was prepared at a stock concentration of 10 mM and Nimodipine and Nitrendipine at 1 mM. Both were diluted to a final concentration of 10 µM. ICA-121431 was prepared as stock solution of 10 mM and used at a final concentration of 1 µM. To prepare stock solutions, Dimethylsulfoxid or ethanol was used as solubilizer if necessary. All stock solutions were kept at −20 °C and diluted to the final concentration with SIF.

### 2.3. Enzyme Immunoassay

iCGRP (immunoreactive calcitonin gene-related peptide) content in the eluates was measured using enzyme immunoassays (Bertin Pharma, Montigny, France) according to our previously described procedures [25,26]. Briefly, the samples were mixed with 5-fold concentrated commercial CGRP enzyme immunoassay buffer (200 µL sample + 50 µL buffer). The buffer consisted of potassium phosphate buffer (0.1 M), NaCl (0.15 M), 0.1% BSA (g/g), 0.01% sodium azide (g/g) and a cocktail of peptidase inhibitors (per mL: leupeptin 20 µg, benzamidin 0.3 mg, pepstatin 2.5 µg, thymostatin 20 µg; plus EDTA 6 mM and THMB 0.36 mg/mL). The enzyme immunoassays were run on 96 well plates, which were photometrically determined using a microplate reader (Dynatech, Channel Islands, UK). In our hands the minimum detection limit was 5 pg/mL. The intra and inter-assay coefficients of variation with repeated measurements were 10–15%.

### 2.4. Data Analysis

All values of CGRP in the eluates were determined as absolute concentrations (pg/mL). For the calculation of the dose-response relationship all values were normalized to the basal CGRP release before stimulation and expressed as fold increase over baseline. Whenever we used absolute concentrations they were baseline subtracted to isolate the effect of P-CTX-1 on CGRP release. In figures and text the values are given as means ± SEM. For all experiments evaluating drug effects, we used matched pair comparisons, i.e., the hindpaw skin of one side served as non-treated control. This procedure allowed the calculation of the variance of the residual CGRP release and the comparisons of the respective drug effect. The paired *t*-test was used for statistical analysis. In all experiments where we used transgenic mice, we obtained two CGRP measurements per mouse, one from each hindpaw skin. The data represent the average of all data points. Data were subjected to analysis with the Grubb’s test for outliers. In addition we calculated a common baseline CGRP value for rat and mouse and discarded single experiments in which the baseline values were higher than 4 times the SD obtained from the calculated average baseline (corresponds to 33.00 pg/mL in the mouse). High baselines usually signify that the skin was damaged during the preparation; then CGRP release occurs during the 30 min washout before the experiment and may deplete the nerve terminals. This concerned 10 experiments in the mouse, none in the rat. Comparison between different groups of experiments was performed with the unpaired *t*-test, one-way or two-way ANOVA. The paired *t*-test was used for comparisons with matched pairs. *P*-values < 0.05 were considered significant. Significance is indicated with * for *p* < 0.05, ** for *p* < 0.01 and *** for *p* < 0.001. The EC_50_ values of P-CTX-1 are defined as the concentrations that elicited 50% of the maximal CGRP release. To determine EC_50_, the experimental data were fitted using a Boltzmann sigmoidal model according to the following equation: $y=\frac{A_{1}-A_{2}}{1+e^{\left(x-x_{0}\right)/dx}}+A_{2}$, where *A*_1_ is the maximum, *A*_2_ is the minimum, *x*_0_ is the half maximal activation and *dx* is the slope factor. Note that the EC_50_ for the mouse was determined from an estimated dose-response curve due to the lack of sufficient concentration points. The purpose of this experiment was to compare sensitivity of mouse and rat hindpaw skin and not to determine the exact EC_50_.

## 3. Results

### 3.1. P-CTX-1-Induced CGRP-Release Is Greater in Mouse Than Rat Skin

In 66 single rat skin flaps the average baseline level was found to be 14 ± 3 pg/mL. In contrast, the mouse baseline levels were more variable and in some instances below the detection limit. In 199 single mouse skin flaps the average baseline level of CGRP was found to be 7 ± 6 pg/mL. The difference between the species was highly significant (*p* = 2.1 × 10^−15^, *t*-test).

**Figure 1:**
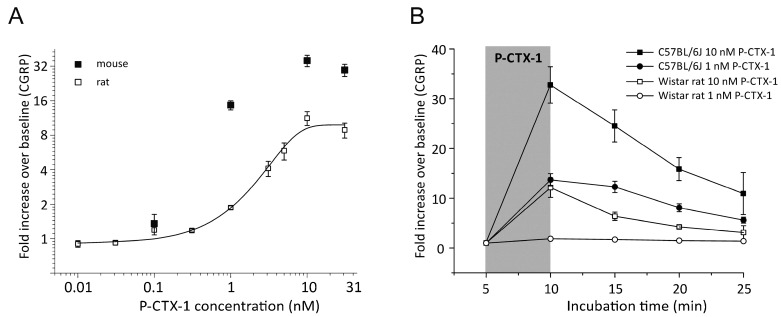
CGRP-release by P-CTX-1 is more effective in mouse than in rat skin. (**A**) Increasing concentrations of P-CTX-1 augmented the iCGRP release dose-dependently in rat and mouse hindpaw skin. The diagram illustrates fold increase of CGRP as compared to baseline. Error bars represent ± SEM; *n* = 4–18 (see text). A Boltzmann sigmoidal model was calculated and yielded ~4 fold increase over baseline at the EC_50_ of 2.7 nM in rat. In the mouse, P-CTX-1 was more effective. The EC_50_ yielded ~13 fold increase of iCGRP. In the rat, 0.31 nM was the effective threshold concentration with iCGRP release being significantly elevated above baseline (*p* = 0.008, *t*-test). (**B**) Release of iCGRP following exposure of rat or mouse hindpaw skin with P-CTX-1 at 1 or 10 nM. Stimulation in mouse tissue was more effective and iCGRP release remained high in the incubation steps after P-CTX-1 was removed. Data are measured as pg/mL and expressed as fold increase over baseline. *Note that the rat concentration response function of panel A uses some of the data displayed in Figure 1G of [8]*.

We first determined a concentration-response relationship in hind paw skin preparations from rats with P-CTX-1 concentrations from 0.01 to 31 nM (Figure 1A). We used all data points which were previously depicted in Figure 1G of [8] and we added additional experiments using 0.031 nM (*n* = 6), and increased the N for the remaining concentrations by *n* = 1–2. In all experiments, stimulation with P-CTX-1 was performed after one initial baseline measurement of CGRP release following 5 min of incubation in SIF. In the subsequent step we measured increasing concentrations of P-CTX-1. The final N included for most concentrations 6 individual experiments: 0.01 nM, 0.031 nM, 0.1 nM, 1 nM (*n* = 18), 3.1 nM, 5 nM (*n* = 4), 10 nM (*n* = 8), and 31 nM (Figure 1A). At a concentration of 0.31 nM, P-CTX-1-induced CGRP release reached 19 ± 1 pg/mL and was significantly higher than the preceding baseline CGRP release (14 ± 2 pg/mL, *n* = 6, *p* = 0.03, *t*-test). From the Boltzmann fit, the EC_50_ was estimated to 2.7 nM P-CTX-1 in rat skin which caused a ~4-fold increase of CGRP release above baseline. We measured CGRP release from mouse hindpaw skin in response to 0.1 (*n* = 2), 1 (*n* = 45), 10 (*n* = 33) and 31 nM (*n* = 4) P-CTX-1 and found a comparable concentration dependency, albeit CGRP-release resulted in a ca. ~13-fold increase above baseline in the mouse at the EC_50_ which was estimated to be around 0.9 nM; thus P-CTX-1 appeared at least 3-fold more effective in the mouse as compared to the rat. At 1 nM the CGRP release reached 14 ± 1 fold (*n* = 45) and at 10 nM CGRP-release was 35 ± 4 fold (*n* = 33) (Figure 1A). This corresponded to total absolute values of 97 ± 9 pg/mL and 136 ± 16 pg/mL, respectively. As illustrated in Figure 1B, the CGRP release remained high and significantly elevated above baseline for all subsequent eluates after the removal of P-CTX-1, especially in the mouse skins. For comparison, in human skin, 1 nM P-CTX-1 caused pain, axon reflex flare, reflex sweating and cold allodynia, the latter one persisting for approximately 4 h after intracutaneous injection of a volume of 20 µL. Concentrations of 0.1 nM caused itch and a smaller, less long-lasting axon reflex flare [8].

### 3.2. P-CTX-1-Induced CGRP Release Requires Extracellular Calcium but Is Not Reduced in Mice Deficient of TRPV1, TRPA1, TRPM8, or TRPC5

**Figure 2:**
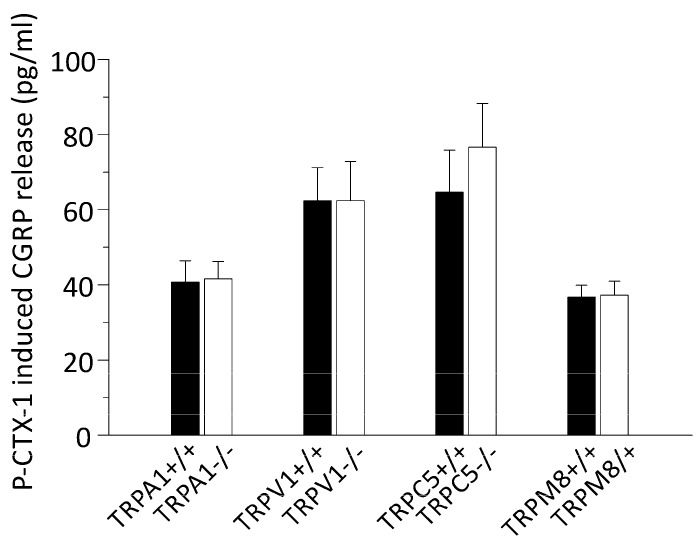
P-CTX-1-induced CGRP release requires extracellular calcium, but is not reduced in mice deficient of the TRP channels TRPV1, TRPA1, TRPM8, or TRPC5. The columns represent 1 nM P-CTX-1-induced iCGRP release in pg/mL as measured in incubation step 2 of the experiment. The values were baseline subtracted and error bars represent ± SEM. White columns show iCGRP-release from transgenic mice and black columns from the respective littermates or C57BL/6J-based control group. No differences were apparent (*p* > 0.05, *t*-test, sample sizes: TRPA1 −/−: *n* = 7 and 8, TRPV1 −/−: *n* = 4 and 5, TRPM8 −/−: *n* = 7 each, TRPC5 −/−: *n* = 6 each).

To elucidate the mechanism by which P-CTX-1 releases CGRP from nerve endings, we assessed P-CTX-1-induced CGRP release in Ca^2+^-free SIF. In these experiments calcium was replaced with 10 mM EGTA. After 5 min of incubation, the skin flaps were exposed to 1 nM P-CTX-1 for 5 min. Upon stimulation with P-CTX-1, hindpaw skins of one side were exposed to normal SIF and responded with an increase of CGRP release of 111 ± 11 pg/mL (*n* = 2) while P-CTX-1 had no effect on the skin flaps of the other side which were incubated in Ca^2+^-free SIF and were CGRP values remained below the detection limit in all incubation steps (*n* = 2) similar to results with Ca^2+^-free SIF in other tissues [12,27,28]. Due to their high calcium permeability, TRP channels are good candidates for mediating P-CTX-1-induced CGRP release which is why we measured CGRP-release in mice deficient of the TRPA1 and the TRPV1 receptor channels, since both TRP channels can be co-localized with CGRP and were previously implicated in P-CTX-1 effects on sensory neurons [6,29]. We additionally tested skin preparations from TRPM8-, and TRPC5-deficient mice, because these channels represent cold transduction channels and may be involved in the temperature misperceptions often reported by ciguatera sufferers [2,30]. For these experiments we calculated the mean CGRP-release of skins from knockouts and compared them to the respective matched controls. The stimulation was performed with 1 nM P-CTX-1 and we found in none of the knockout mice any decrease of the P-CTX-1-induced CGRP release. The P-CTX-1-induced CGRP release was in TRPA1 −/− 42 ± 5 pg/mL (vs. 41 ± 6 pg/mL in controls), in TRPV1 −/− 62 ± 10 pg/mL (vs. 62 ± 9 pg/mL), in TRPM8 −/− 37 ± 4 pg/mL (vs. 37 ± 3 pg/mL), and in TRPC5 −/− 77 ± 12 pg/mL (vs. 65 ± 11 pg/mL; skins from *n* = 4–8 mice; Figure 2).

### 3.3. P-CTX-1-Induced CGRP Release Requires Extracellular Sodium and Activation of VGSCs

**Figure 3:**
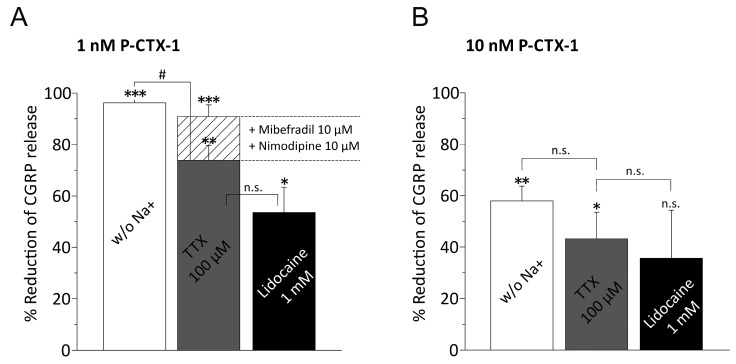
CGRP release by P-CTX-1 requires extracellular sodium via VGSCs activation and induces VGCC activation. The columns represent the reduction of the P-CTX-1-induced iCGRP release by extracellular solution deprived of sodium ions and the respective blockers TTX 100 µM or Lidocaine 1 mM in murine skin. The values were calculated from baseline subtracted iCGRP release in pg/mL as measured in incubation step 2 and represent a matched pair comparison where one skin side was treated with drug and the other served as matched control. Statistical comparisons were performed with the respective *t*-tests for paired groups (asterisks) or ANOVA (unpaired groups, hashtags where applicable). Significance is indicated with * or # for *p* < 0.05, ** for *p* < 0.01 and *** for *p* < 0.001; *n.s.* relates to no significant difference. (**A**) Percent reduction of iCGRP-release induced with 1 nM P-CTX-1. Sample sizes: sodium-free solution: *n* = 4 (matched pairs, *p* = 1.9 × 10^−5^); 100 µM TTX: 8 pairs (*p* = 0.001); 1 mM lidocaine: *n* = 4 pairs (*p* = 0.03). The matched-pair comparison of the reduction of P-CTX-1-induced CGRP-release by TTX 100 µM alone (grey column) and the combination of TTX 100 µM with T- and L-type VGCC blockers mibefradil 10 µM and nimodipine 10 µM is calculated as reduction of the residual iCGRP release after TTX-block and is superimposed and visualized as the bar filled with striped pattern. The residual iCGRP release after TTX-block is reduced by VGCC block by additional 17% (*n* = 14 matched pairs, *p* = 0.008, paired *t*-test). (**B**) Percent reduction of iCGRP-release induced with 10 nM P-CTX-1. Sample sizes: sodium-free solution: *n* = 11 (matched pairs, *p* = 0.002); 100 µM TTX: *n* = 6 (*p* = 0.047); 1 mM lidocaine: *n* = 10 (*p* = 0.06). Notably increasing concentrations of P-CTX-1 reduce the effect of both sodium channel block and deprivation of extracellular sodium (see text).

Ciguatoxin exerts its neuronal effects via activation of TTXr and TTXs VGSC subtypes [3,4,5,6]. Therefore we next assessed to which extent extracellular sodium is required to mediate the P-CTX-1 effect. In these experiments we used choline chloride and HEPES to replace sodium (see methods). In sodium free solution, 1 nM P-CTX-1 was ineffective and the CGRP-release appeared reduced to 4 ± 1 pg/mL (*n* = 4 matched pairs) above baseline, which was 96 ± 2% less than in the matched contralateral hindpaw skin (112 ± 21 pg/mL; *n* = 4, *p* = 1.9 × 10^−5^, paired *t*-test). With 10 nM P-CTX-1, the CGRP-release was 25 ± 5 pg/mL in comparison to the contralateral matched controls (63 ± 14 pg/mL; *n* = 11 matched pairs; *p* = 0.002, paired *t*-test) which corresponded to an average reduction by 58 ± 6% (Figure 3A,B).

We next tested whether VGSC blockers lidocaine and TTX had effects equivalent to the sodium free solution. We first tested in rat skin the blocking effect of 1 µM TTX on 1 and 3.1 nM P-CTX-1 (*n* = 6 matched pairs each) and of 10 µM TTX on 1 nM P-CTX-1 (*n* = 3 matched pairs), but found no clear indication for a reduction of CGRP-release. In the next experiments we used mouse preparations and TTX at a concentration of 100 µM, which should not leave any TTXr or TTXs VGSC subtypes unblocked. In these experiments we induced CGRP-release with 1 nM and 10 nM P-CTX-1. We applied 100 µM TTX in the second incubation step and combined P-CTX-1 and TTX in the third incubation step. Using 1 nM P-CTX-1 we found CGRP-release reduced to 22 ± 8 pg/mL (*n* = 8 matched pairs), which corresponded to 74 ± 6% CGRP less than in the matched contralateral hindpaw skin sides (81 ± 19 pg/mL; *n* = 8; *p* = 0.0012, paired *t*-test). Similar to the experiments with sodium free solution, with 10 nM P-CTX-1, TTX was less effective and the P-CTX-1-induced CGRP-release blocked by 43 ± 10% which corresponded to 113 ± 26 pg/mL on the TTX-treated side and 218 ± 35 pg/mL on the contralateral matched controls (*n* = 6 per group; *p* = 0.048, paired *t*-test; Figure 3A,B). Remarkably, the levels of reduction achieved with sodium free external solution compared to 100 µM TTX were significantly different in case of 1 nM P-CTX-1 (*p* = 0.033, one-way ANOVA with LSD post-hoc test), but this difference vanished at 10 nM (*p* = 0.48, one way-ANOVA with LSD post-hoc test).

Next, we compared these results to 1 mM Lidocaine and induced CGRP-release with 1 nM and 10 nM P-CTX-1. With 1 nM P-CTX-1 we found CGRP-release blocked by 53 ± 10% (*n* = 4; *p* = 0.03, *t*-test) and with 10 nM P-CTX-1 the blocking effect was reduced to 36 ± 19% and the difference between untreated side and lidocaine-treated skins was no longer significant (99 ± 13 pg/m and 59 ± 14 pg/m; *n* = 10 matched pairs; *p* = 0.06, paired *t*-test). Similar to sodium free solution and 100 µM TTX, blocking sodium channels with lidocaine lost some of its effect compared to sodium free with increasing P-CTX-1 concentration; *p* = 0.002 for 1 nM P-CTX-1, and *p* = 0.43 for 10 nM P-CTX-1 (one-way ANOVA with LSD post-hoc test; Figure 3A,B).

Taken together, with the increased concentration of P-CTX-1, the effect of sodium deprivation and also of 100 µM TTX and 1 mM lidocaine was significantly reduced in contrast to 1 nM P-CTX-1 (*p* = 0.013, two-way ANOVA between 1 and 10 nM P-CTX-1).

Lidocaine, although applied at 1 mM, appeared weaker in its blocking effect than the VGSC inhibitor TTX at 100 µM. We tested whether lidocaine’s blocking effect of the P-CTX-1-induced CGRP-release was specific for its action on VGSC. For this purpose we induced CGRP release with 60 mM KCl, but in this experiment lidocaine had no blocking effect (KCl: 104 ± 27 pg/m and KCl + 1 mM lidocaine: 91 ± 22 pg/m; *n* = 10 matched pairs; *p* = 0.2, *t*-test). Thus, VGCC or the vesicle release machinery were not impaired by 1 mM lidocaine in rat or mouse skin [31].

### 3.4. P-CTX-1-Induced VGSC Opening Triggers Activation of L- and T-Type VGCC

Both high-threshold-activated L- and low-threshold T-type VGCC are present in nociceptive CGRP-expressing fibers and contribute significantly to CGRP release from axons, especially in response to high KCl, which induces a slow depolarization [32]. Mibefradil blocks T-type VGCCs, and L-type channels are blocked by nimodipine. We applied both blockers in combination with TTX 100 µM at concentrations of 10 µM. Like the other series, these experiments were performed as matched pair comparisons, here, to the application of TTX 100 µM alone. Specifically, one hindpaw was incubated with 100 µM TTX in the second step and combined P-CTX-1 (1 nM) and TTX (100 µM) were applied in the third incubation step. The contralateral paws received, in step 2 in addition to TTX 100 µM, 10 µM of each mibefradil and nimodipine and in the third step the three blocker cocktail was applied in combination with 1 nM P-CTX-1. The incubation in step 4 contained only mibefradil and nimodipine. We found that the VGCC-blockers reduced the TTX blocked fraction of the CGRP release by additional 65 ± 7% or rather it extended the TTX-block by additional 17% (Figure 3A). In this experiment, the CGRP release appeared reduced from 54 ± 8 pg/mL to 16 ± 7 pg/mL by VGCC block (*n* = 12 matched pairs; *p* = 0.008, paired *t*-test). From these experiments, the combination of VGSC and L- and T-type VGCC block abolished the P-CTX-1-induced CGRP-release to almost a level as achieved with sodium deprivation (Figure 3A). Mibefradil 10 µM alone before and then in combination with the P-CTX-1 stimulation was ineffective in changing the induced CGRP release (*n* = 12, not shown). Similarly, in a comparable experiment, L-type blocker nitrendipine 25 µM (*n* = 8, not shown) did not change the P-CTX-1-induced CGRP-release.

### 3.5. Distinct VGSC Subtypes Mediate the P-CTX-1-Induced CGRP Release

To elucidate the specific VGSC subtypes that initiate the CGRP release we used transgenic mice lacking either of the sodium channels Na_V_1.7 (floxed Na_V_1.7—Advillin-Cre-null mutant mice), Na_V_1.8 or Na_V_1.9, which co-localize in peptidergic C-fibers [33]. We also used the Na_V_1.1/1.3 specific sodium channel blocker ICA-121431 [16] to investigate the role of Na_V_1.1, which was recently reported to be expressed in a specific nociceptor subpopulation presumably of Aδ-fiber type including CGRP positive neurons [34]. In our previous publication we described that P-CTX-1 led to profound activation and sensitization to cold of Aδ-fibers and induced extraordinarily high firing rates [6]. The expression of TTXs Na_V_1.3 occurs mainly in early developmental stages and is very low in healthy adult DRG.

**Figure 4:**
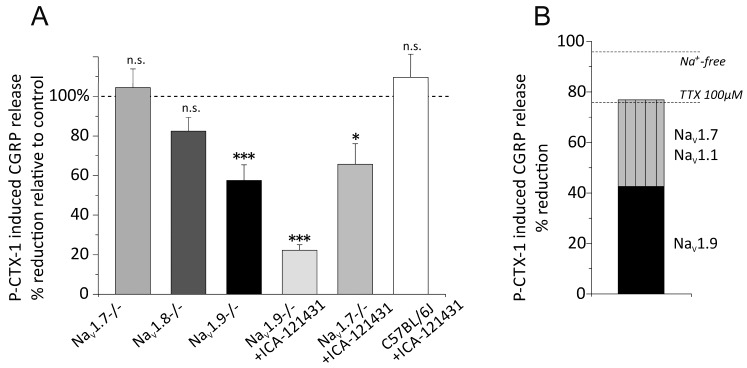
P-CTX-1-induced CGRP release is mediated by three distinct VGSC subtypes. The columns represent P-CTX-1-induced iCGRP release in pg/mL as measured in P-CTX-1 incubation step of the experiment. The values were baseline subtracted and error bars represent mean ± SEM. Statistical comparisons were performed with the *t*-test. Significance is indicated with * for *p* < 0.05, ** for *p* < 0.01 and *** for *p* < 0.001; *n.s.* relates to no significant difference. (**A**) Columns show % reduction of iCGRP-release from transgenic mice compared to the respective littermates or C57BL/6J control group. The sample sizes were: Na_V_1.8 −/−: *n* = 8, Na_V_1.7 −/−: *n* = 6, Na_V_1.9 −/−: *n* = 8, Na_V_1.9 −/− + ICA-121431 (Na_V_1.1 blocker): *n* = 5, Na_V_1.7 −/− + ICA-121431: *n* = 4, C57BL/6J + ICA-121431: *n* = 4. Significant reduction of P-CTX-1-induced iCGRP release occurred in Na_V_1.9 −/− and amounted to 42% and 1 µM ICA-121431 further increased the block to 78%. In Na_V_1.7 −/− 1 µM ICA-121431 reduced CGRP release by 34% (*p* = 0.0002 and *p* = 0.03, respectively, indicated by asterisks). (**B**) Schematic calculation of the major contributors to the 1 nM P-CTX-1-induced CGRP release. Dashed lines illustrate the reducing effect of sodium deprivation and the TTX-blocked fraction. The VGSC contribution is composed of Na_V_1.9 (42%) and Na_V_1.7 + Na_V_1.1 (34%). Note that both may contain some VGCC effect and also other VGSC subtypes might contribute with minor fraction.

Although Na_V_1.8-containing pathways are a key component in the pathognomonic symptom of cold allodynia, we found that absence of Na_V_1.8 had no effect on the P-CTX-1-induced CGRP release. In Na_V_1.8 −/− the 1 nM P-CTX-1-induced CGRP release was 57 ± 5 pg/mL which was insignificantly less than in the littermate skins which released 69 ± 8 pg/mL (18% difference; *n* = 8 mice; *p* = 0.2, *t*-test; Figure 4). Unexpectedly, the absence of Na_V_1.7 also failed to cause any reduction of the CGRP release which was 72 ± 7 pg/mL and corresponded to 69 ± 7 pg/mL in the littermates (5% difference; *n* = 6 mice; *p* = 0.7, *t*-test; Figure 4). In Na_V_1.9 −/− mice, we found the 1 nM P-CTX-1-induced CGRP release significantly reduced. The release was 72 ± 10 pg/mL and this corresponded to 42% less than the release of 126 ± 9 pg/mL in the littermates (*n* = 8 mice per group; *p* = 0.002, *t*-test). To test whether CGRP release is impaired in Na_V_1.9 −/−, we used KCl 60 mM as a stimulus to release CGRP independent of VGSC activation and found that both genotypes responded similarly to the KCl (183 ± 32 pg/mL and 153 ± 40 pg/mL, respectively, *n* = 4 mice). Thus, apparently P-CTX-1 has clear effects on the TTXr Na_V_1.9 channels and this subtype accounts for half of the VGSC-mediated effect. We next tested the Na_V_1.1 blocker ICA-121431 at 1 µM in Na_V_1.7-deficient mice, and found that absence and block of these two VGSC subtypes, respectively, led to a significant reduction of the P-CTX-1-induced CGRP-release. In this experiment, the 1 µM ICA-121431-blocked 1 nM P-CTX-1-induced CGRP-release in the Na_V_1.7 +/+ skins was 76 ± 8 pg/mL, similar to the other congenic mouse strains, and this appeared reduced by 34% to 50 ± 8 pg/mL in ICA-121431-blocked Na_V_1.7 −/− skins (*n* = 8 mice per group; *p* = 0.03, *t*-test). Similarly, application of 1 µM ICA-121431 to the skins of Na_V_1.9 −/− led to further decrease of CGRP-release to 28 ± 4 pg/mL and left 22% residual CGRP release compared to the Na_V_1.9 +/+ group. The effect of 1 µM ICA-121431 on 1 nM P-CTX-1-induced CGRP release in wild-type C57BL/6J mice was indifferent from untreated controls (Figure 4A, right column). We also tested the effect of 1 µM ICA-121431 on Na_V_1.8-deficient skins, but found no reduction of the 1 nM P-CTX-1-induced CGRP release.

## 4. Discussion

Our findings illustrate that P-CTX-1 leads to potent CGRP release in a concentration-dependent manner in rat and mouse skin. The effective threshold concentration for CGRP release in the rat was 0.31 nM and the EC_50_ was 2.7 nM. In the mouse, the EC_50_ was estimated to 0.9 nM; nevertheless at the EC_50_, the CGRP release was augmented ca. 4-fold in the rat, while it was increased by ca. 13-fold in the mouse. At saturating concentrations this reached a maximum of 12- and 33-fold, respectively. This difference may relate to mouse skin being thinner and its superficial nerve endings being more efficiently exposed than the thicker rat skin [26]. In addition, in the mouse CGRP can pass the epineurium in contrast to the rat and the diffusion barrier for drugs is reduced [35]. TTX for example requires between 120 and 180 seconds in the mouse to block nerve endings, while in the rat 20–40 minutes are observed [36,37]. Remarkably, ciguatoxins are the most potent drugs in inducing CGRP release identified, with 1 nM P-CTX-1 releasing 98 ± 9 pg/mL from mouse hindpaw skin. In a previously established co-culture of rat DRGs with human keratinocytes, P-CTX-2 was found to release even more CGRP; at 1 nM on average 300 pg/mL were released and with 10 nM the CGRP release was even higher and it augmented further over time [15]; this is particularly surprising, because P-CTX-2 is 9-times less potent than P-CTX-1 in mice [24]. Most likely the co-culture model contains higher amounts of CGRP *per se* or is a more sensitive assay for measuring CGRP release. In the mouse skin, 10 nM was already the saturation concentration and induced a 33-fold increase of CGRP over baseline values which corresponded to 135 ± 16 pg/mL CGRP. The potency of CTXs is quite remarkable and its large effect on CGRP release could be of great importance for the clinical symptomatology of ciguatera.

P-CTX-1-induced neuropeptide release requires calcium-dependent exocytosis, because deprivation of extracellular Ca^2+^ or Na^+^ led to complete inhibition of the stimulated release and the release is blocked by specific blockers of VGSCs and VGCCs. Release of calcium from intracellular stores, cytotoxicity or pore-forming effects as they occur with the bacterial toxin ionomycin do not contribute to the P-CTX-1 effect [38,39]. This is in line with previous studies that illustrated neurosecretory effects of CTXs from chromaffin cells and synapses [40,41]. TRPA1, which we found earlier to be a prominent molecular marker of a population of CGRP-expressing sensory neurons, which are strongly affected by P-CTX-1 [6], would have been a promising candidate to mediate the increase in intracellular calcium in these neurons due to its high calcium conductance [42]. Similarly important, the CTX-related gambierol was identified to exert an allosteric effect on the TRPV1 receptor via an acting site related to the intracellular binding site of capsaicin [29]. Nevertheless our experiments with the null-mutant mice demonstrated that none of the major thermosensory TRP-channels contributed to the P-CTX-1-mediated effects on CGRP release, at least not with 1 nM. The lack of a TRPM8 effect is not surprising because it is sparsely co-expressed with CGRP [43]. As for TRPC5, data are not available but its expression concerns about 30% of small-diameter DRGs in the mouse, and little co-expression with TRPM8 occurs [44].

The primary targets of CTXs are TTXs and TTXr VGSC [3,4,5,6]. Similar to a previous report from the DRG-keratinocyte co-culture [15], we found that only a high concentration of TTX (100 µM), inhibiting all VGSC, had a blocking effect on the skin preparations, but the effect was still less as compared to sodium free solution (74% reduction by TTX as compared to a 96% reduction in sodium free solution that should safely prevent any depolarization upon P-CTX-1). The effect of lidocaine was a 53% reduction, statistically indifferent from TTX 100 µM. The neuronal afferents innervating the hindpaw skin comprise sensory saphenous nerves, and CGRP is expressed in ~40% of Aδ-fibers and 50% of C-fibers [45,46] which contribute to the release by depolarizing sodium channel subtypes. In our experiments, the major contributor and only TTXr subtype was Na_V_1.9. Its absence reduced CGRP release by 42%. The lack of a difference between Na_V_1.8 and littermates indicated that Na_V_1.8 does not contribute to the CGRP-release despite its substantial contribution to the action potentials in nociceptors [47,48]. Na_V_1.8 is found in 85% of the C-fiber nociceptors [49] and, even more, our previous results identified Na_V_1.8 as a major contributor to cold allodynia caused by P-CTX-1 in mice via peptidergic nociceptors [6]. Nevertheless, both Na_V_1.8 and Na_V_1.9 co-localize with peripherin and have a considerable overlap in peptidergic neurons [33,50,51]. The Na_V_1.9-mediated sodium current produces a persistent non-inactivating current, which is important in the regulation of C-fiber excitability. This current allows sub-threshold long-lasting depolarization and oscillatory bursting discharge [52,53], which may be relevant for the oscillatory depolarization caused by P-CTX-1 [54]. Importantly, the CGRP release itself was not impaired in the Na_V_1.9 −/− mice since KCl-induced CGRP release was unaffected.

The relevant TTXs VGSC candidates potentially mediating the remaining CGRP-release included Na_V_1.7, Na_V_1.1 and Na_V_1.6, because Na_V_1.3, as well as Na_V_1.2 are undetectable in adult DGR neurons [50] and a recent report has confirmed the presence of Na_V_1.1 in a subpopulation of peptidergic Aδ-fibers with in situ hybridization [34]. The large concentration of TTX required to effectively reducing CGRP release may indicate that it is only caused by P-CTX-1 effects on TTXr VGSC subtypes. However, our experiment of combining block of Na_V_1.7 deletion or Na_V_1.9 deletion with Na_V_1.1 block indicates that more likely simultaneous activation of TTXs and TTXr VGSC subtypes and subsequent VGCC activation mediate the P-CTX-1-induced CGRP release. The Na_V_1.9 deletion reduced the P-CTX-1 response by 42%, additional block of Na_V_1.1 reduced it further and left 22% residual CGRP-release. In addition the pharmacological block of Na_V_1.1 in Na_V_1.7 knockouts reached 34% reduction, together with the effect of Na_V_1.9 these three VGSC subtypes would account for about 76% which is the degree of inhibition achieved with 100 µM TTX. Further inhibition of P-CTX-1-induced CGRP release by about 17% was achieved by combining TTX with the VGCC blockers. Thus, the inhibitory effect of extracellular sodium depletion was also reached by combining the pharmacological VGSC and VGCC channel antagonists. However, with 1nM P-CTX-1 the VGCC blockers mibefradil and nitrendipine applied alone, i.e., without TTX 100 µM, had no measurable effect on the CGRP release. Likely the effect of each VGCC subtype is too small to be detected separately.

Although activation by P-CTX-1 of VGSCs (Na_V_1.9, Na_V_1.7 and Na_V_1.1) and secondary activation of VGCCs seems to account for most of the CGRP-releasing effect of 1 nM P-CTX-1, the 10 nM, saturating concentration of the toxin obviously involved further mechanisms that were partly (ca. 40%) independent of depolarizing sodium influx and less effectively blocked by 100 µM TTX or 1mM lidocaine. The CTX-related toxin gambierol of *Gambierdiscus toxicus* binds to voltage-gated potassium channels (VGKC) and thereby leads to block and induces neuronal Ca^2+^ oscillations with an EC_50_ of 18 nM [55]. Direct effects of gambierol on particular subtypes were also measured and occurred at EC_50_ between 34 and 64 nm [56]. Block of VGKC was previously found for CTX and occurred at a concentration of 2–20 nM [57]. It is likely that VGKC become directly inhibited, contributing to the CGRP release. TRP channels, such as TRPV1 were also modulated by gambierol, but the effects were not direct and involved allosteric modulation, which occurred with an EC_50_ of 600 nm [29]. Therefore it is less likely that 10 nM CTX would open TRPV1 or TRPA1 directly.

Although much later clarified, the potent vasodilatory action of CGRP was discovered in 1901 when Bayliss described the antidromic vasodilatation evoked by electrical stimulation of spinal dorsal roots [58]. This phenomenon obviously depends on the generation and propagation of action potentials, like the sensory phenomena associated with Ciguatera depend on the transmission by action potentials. When CGRP release evoked by electrical (saphenous) nerve stimulation of isolated rat skin was first measured, a threefold increase over baseline was achieved by 1200 pulses during 5 min at 4/s [59]. In major contrast, P-CTX-1 could achieve a 12-fold increase of CGRP release from rat hairy hindpaw skin (at saturating concentration) and 33-fold (Figure 1) from mouse hairy hindpaw skin, suggesting that action potentials are much less efficient in raising intraaxonal calcium and evoking neuropeptide exocytosis than the sustained depolarization caused by P-CTX-1. This may finally account for the major role in our results of the non-inactivating Na_V_1.9 in contrast to the more or less rapidly inactivating Na_V_1.7 and Na_V_1.8 that are decisive as action potential generators for the sensory symptoms but not for the neurogenic inflammation caused by ciguatoxins.
